# A decade of pig genome sequencing: a window on pig domestication and evolution

**DOI:** 10.1186/s12711-016-0204-2

**Published:** 2016-03-29

**Authors:** Martien A. M. Groenen

**Affiliations:** Animal Breeding and Genomics Centre, Wageningen University, Droevendaalsesteeg 1, 6708 PB Wageningen, The Netherlands

## Abstract

Insight into how genomes change and adapt due to selection addresses key questions in evolutionary biology and in domestication of animals and plants by humans. In that regard, the pig and its close relatives found in Africa and Eurasia represent an excellent group of species that enables studies of the effect of both natural and human-mediated selection on the genome. The recent completion of the draft genome sequence of a domestic pig and the development of next-generation sequencing technology during the past decade have created unprecedented possibilities to address these questions in great detail. In this paper, I review recent whole-genome sequencing studies in the pig and closely-related species that provide insight into the demography, admixture and selection of these species and, in particular, how domestication and subsequent selection of *Sus scrofa* have shaped the genomes of these animals.

## Background

The domestic pig (*Sus scrofa*) is a member of the family of Suidae, a group of pig species from the order of Cetartiodactyla that originated some 20 to 30 million years ago (Mya) [1, 2]. Of this family, *S. scrofa* (wild boars and domestic pigs) is the only species that was domesticated [2]. Sequencing its genome was initiated with the establishment of the Swine Genome Sequencing Consortium (SGSC) in September 2003 [3], following the successful generation of genetic [4] and physical [5] maps for the pig. The strategy by the SGSC was based on hierarchical shotgun Sanger sequencing of bacterial artificial chromosome (BAC) clones representing a minimal tile path across the genome [6], which was supplemented at a later stage with Illumina next-generation sequencing data [7]. These efforts resulted in the assembly and publication of a draft reference genome sequence of *S. scrofa* in 2012 [8]. In addition to this reference genome sequence, which was derived from a female Duroc pig, the SGSC described the genome sequence of another 48 pigs from a variety of breeds and wild boars [8]. Moreover, an independent genome assembly of a Chinese Wuzhishan minipig, based on Illumina short reads, was published simultaneously [9], followed by additional de novo assembled genomes of a Göttingen minipig [10] and a Tibetan wild boar [11] in 2013. Since then, the genomes of hundreds of individual pigs have been re-sequenced to study genome variation, evolution, and selection in this species [11–25], and currently around 350 complete genomes are publically available (Table 1).

**Table 1:** Pig whole-genome resequencing: overview of pig short read Illumina sequences deposited in the European Nucleotide Archive (1-9-2015)

Accession	Species	Pig breeds/populations	Publications	Number of individuals
PRJEB1683	Ssc, Svr, Sba, Sce, Scb, Paf	EWB, AWB, DU, HA, PI, LR, LW, XI, JQ, MS	[8, 12, 14]	77
PRJNA144099	Ssc	WZ	[9]	1
PRJNA41185	Ssc	DU	–	1
PRJNA176189	Ssc	GM	[10]	1
PRJNA231897	Ssc	RC	–	6
PRJNA186497	Ssc	AWB, TWB, PZ, WJ, YN, NJ, JH	[11]	49
PRJNA238851	Ssc	TC	[23]	5
PRJNA260763	Ssc	DU, LR, YM, KWB, LW	[18, 19]	70
PRJEB9115	Ssc	DU^a^	–	1
PRJNA213179	Ssc	AWB, BX, EH, HT, LA, LU, MI, GA, SC, TP, YN, WZ	[16]	69
PRJNA221763	Ssc	BK	[25]	3
PRJNA239399	Ssc	MA, DU	[15]	4
PRJNA190683	Ssc	IB	–	1
PRJNA255085	Ssc	EWB, GC, IB, MP	[22]	4
PRJEB9326	Ssc, Scb	PI	[24]	18
PRJEB9922	Ssc, Svr	EWB, ASW, WS, ZA, LS, AS, BB, BK, BS, CM, CS, CA, CT, GO, HA, LB, LI, MA, MW, IB, NS, TA, RE, JQ, XI	[20]	102
PRJNA281548	Ssc	BK	[21]	10

Ssc, *Sus scrofa;* Svr, *Sus verrucosus*; Sba, *Sus barbatus*; Sce, *Sus celebensis*; Scb, *Sus cebifrons*; Paf, *Phacochoerus africanus*, AS, Angler Sattleschwein; AWB, Asian wild boar; BB, Bunte Bentheimer; BK, Berkshire; BS, British Saddleback; BX, Bamaxiang; CA, Calabrese; CM, Chato Murciano; CS, Cinta Senese; CT, Casertana; DU, Duroc; EH, Erhualian; EWB, European wild boar; GA, Gansu; GC, Guatemala Creole pig; GM, Göttingen minipig; GO, Gloucester Old Spot; HA, Hampshire; HT, Hetao; IB, Iberian; JH, Jinhua; JQ, Jiangquhai; KWB, Korean wild boar; LA, Laiwu; LB, Large Black; LE, Leicoma; LI, Linderodsvin; LR, Landrace; LS, Leping Spotted; LU, Luchuan; MI, Min; LW, Large White; MA, Mangalica; MP, 16th century pig; MS, Meishan; MW, Middle White; NJ, Neijiang; NS, Nera Siciliana; PI, Pietrain; PZ, Penzhou; RC, Rongchang; RE, Retinto; SC, Sichuan; TA, Tamworth; TC, Tongcheng; TP, Tibetan pig; TWB, Tibetan wild boar; WJ, Wujin; WS, Wannan Spotted; WZ, Wuzhishan; XI, Xiang; YM, Yucatan miniature pig; YN, Yannan; ZA, Zang

^a^Short read sequences of the Duroc pig on which the pig reference genome assembly [8] is based

The Suidae family represents a diverse group of species that comprises 15 to 17 extant species that are grouped into five genera [1, 2]. Although currently a de novo assembled genome is only available for *S. scrofa*, their close evolutionary relationship to *S. scrofa* allows the use of its reference genome [8] for analysis of these other species as well. Consequently, the genomes of 10 other species of this family have been sequenced and studied by aligning the sequences against the pig reference genome [26, 27]. Particular care has to be taken using such across-species sequence alignments, since aligning sequences against the genome of another species can result in underestimation of sequence variability. Nevertheless, this can be controlled by carefully choosing the sequence alignment program and the genotype-calling algorithm [27].

## Review

### Suid speciation

**Fig. 1:**
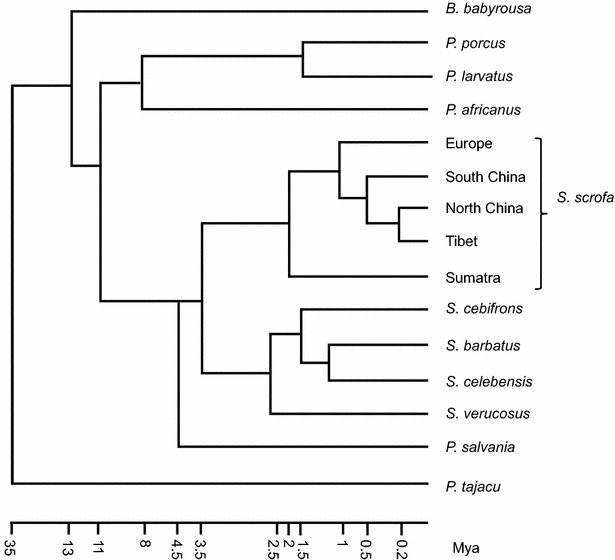
Schematic phylogenetic tree. The phylogeny diagram summarises the results of phylogenetic studies based on whole-genome sequence data [26–28]. Timing is derived from the estimates described in [26–28, 30]

 Members of the five genera of the Suidae family can be found across Africa, Europe, and Asia, and over the past decade the genomes of several of these species have been sequenced. Among the suids found in Africa, the genomes of three species from two different genera have been sequenced [27]: *Phacochoerus africanus* (common warthog), *Potamochoerus porcus* (red river hog), and *Potamochoerus larvatus* (bush pig), while the genomes of seven species from three genera have been sequenced from Suids in Asia [five *Sus* sp. [26], *Babyrousa babyrussa* [27] and *Porcula salvania* (pygmy hog; MAM Groenen, unpublished results)]. The largest variety of pig species is found on the Islands of South East Asia (ISEA), which is likely a result of the large number of isolated islands separated by a shallow sea that during glacial periods and low sea levels allowed them to be connected by land bridges [26]. Currently, six different species from this area have been sequenced [12, 26, 27]: *S. scrofa* (from Sumatra), *Sus verrucosus* (Javan warty pig), *Sus barbatus* (bearded pig), *Sus celebensis* (Sulawesi warty pig), *Sus cebifrons* (Visayan warty pig), and *B. babyrussa* (pig-deer). Apart from the pygmy hog from Northern India, all the other wild pigs from Asia that have been sequenced are considered a single species (*S. scrofa*), although phylogenetic analysis based on their sequenced genomes shows that several of these populations diverged more than a million years ago (Fig. 1) [28, 29]. The availability of complete genome sequences of these species and sub-species allowed the reconstruction of a well-supported phylogeny (Fig. 1) that shows that some of the *S. scrofa* populations in Eurasia diverged at around the same time as some of the *Sus* species found in ISEA. In spite of the deep phylogenetic split between species of the genus *Sus*, extensive gene flow has occurred between many of these species through natural dispersal as a result of land bridges during glacial periods [26, 30]. To date, there are no detailed analyses of selective sweeps during the speciation of these species, but a comparison of copy number variation (CNV) between the Asian suids [31] showed that olfactory receptor genes and immune-related genes are among the most rapidly evolving genes. The latter likely reflects adaptation to different pathogens in different environments. It has been suggested [32] that the rapid evolution of the olfactory receptor gene repertoire was not only an adaptation to the new environment but also might have acted as a species barrier by affecting mate choice.

### Dispersal and divergence of *S. scrofa* populations across Eurasia

*Sus scrofa* (hereafter referred to as wild boar or as pig in the case of the domesticated populations) originated in Southeast Asia some 3 to 4 Mya and over the past one million years colonized almost the entire Eurasian mainland. Wild boars are also found on the Northern parts of Sumatra, although this population diverged from the Eurasian wild boar some 1.5 to 2 Mya [26, 27] (Fig. 1). The European and Asian wild boar populations diverged around 1 Mya [8, 26], resulting in very different minor allele frequencies at millions of genomic locations and over a million locations that are fixed for alternative alleles [8]. In agreement with the CNV analysis of the other suids [31], genes involved in sensory perception, immunity, and host defence were among the most rapidly evolving genes [32]. A selective sweep analysis for the split between European and Asian wild boars, using a method that was originally developed to study human Neanderthal divergence and that is based on identification of regions that share fewer derived alleles [33], identified 251 putative selected regions [8]. Surprisingly, these regions displayed an over-representation of genes involved in RNA splicing and RNA processing, suggesting changes in gene expression and gene regulation during adaptation to novel habitats while the species expanded across Eurasia.

Within Asia, there is a clear split between wild boar populations in the North (North China, Tibet, Japan) and the South (South China), with an estimated divergence time of around 0.5 Mya [26, 27]. A selective sweep analysis between these populations (including both wild boar and domestic breeds from these regions), using locus-specific branch-length analyses [34], indicated that genes involved in biological processes that contribute to maintenance of thermostatic status during heat or cold stress, such as hair growth (*DCAF17*) and blood circulation (*VPS13A*), have been under divergent selection [16]. These results confirmed earlier studies on a de novo assembled genome of a Tibetan wild boar [11]. This latter study also showed possible selection in Tibetan wild boars for adaptation to high altitude. Examples of this adaptation are genes with vitamin B6 binding activity (*ALB*, *SPTLC2*, and *GLDC*) and genes related to hypoxia (*ALB*, *ECE1*, *GNG2*, and *PIK3C2G*). It was also suggested that the Tibetan wild boar exhibited extensive lineage-dependent gene expansion and contraction (e.g. the olfactory receptor genes) [11]. However, these observations are most likely mainly the result of the draft status of the pig reference genome [8, 35] and collapses of repetitive and duplicated regions in the next-generation sequencing-based genome assembly of the Tibetan wild boar [28, 36].

The X chromosome of European and Asian pigs and wild boars shows a remarkable 48-Mb large region of homozygosity [16, 37] that overlaps with a region that shows almost no recombination [38]. Effectively, this region exhibits two different haplotypes, one found in European and North Chinese pigs and wild boar, and one in pigs and wild boar from South China. In addition, a third haplotype is observed in some North Chinese pigs [16] and Meishan pigs (HJ Megens and MAM Groenen, unpublished results), which appears to be a recombinant haplotype between the first two haplotypes. In fact, the South Chinese haplotype clusters with haplotypes observed in the other *Sus* species from ISEA, which indicates that it may have originated from an admixture event between South Chinese wild boar and another, now extinct, suid. One explanation of the current distribution of this haplotype is that it provided an advantage in Northern latitudes and therefore has swept to fixation, and was subsequently transmitted to European wild boar populations as a result of migrations across Eurasia [16]. Gene flow was also observed between *S. scrofa* and other *Sus* species on ISEA and, although it was originally attributed to human-mediated dispersal of pigs across this area [26], subsequent analyses indicated that it was the result of natural dispersal of these pigs in South East Asia [30]. Furthermore, admixture between *S. scrofa* and *S. verrucosus* was shown to be predominantly the result of gene flow from *S. verrucosus* into *S. scrofa*, mostly likely between 0.5 and 1 Mya [30].

### Suid demography

An interesting feature of the availability of whole-genome sequence data from a diploid species’ individual is that it allows the inference of its demography up to several hundreds of thousands generations ago (e.g. by using pairwise sequential Markovian coalescence; PSMC [39]). Using PSMC and assuming an equal generation time of 5 years and the same mutation rate of 2 × 10^−8^ for all *Sus* species, indicated that wild boars from Eurasia and most suids from ISEA experienced a strong bottleneck during the Pleistocene period [8, 26]. These declines in population size are in agreement with the reduction of temperature during this period, which resulted in reduced overall forest cover, which is the natural habitat for these species. For many of the species, in particular *S. scrofa*, the population sizes reached their minimum during the last glacial maximum [8], around 20 thousand years ago (Kya). Pig populations in Europe were the most affected by this climate change and it has been proposed that wild boar populations in Europe retreated into three refugia during that period, i.e. Iberia, Italy and the Balkans [40]. In Asia, wild boar populations in Northern China and Tibet were more affected than populations in South China [26, 28]. Interestingly, the population size of *S. celebensis* on Sulawesi Island appears to have increased dramatically towards the end of the interglacial period that preceded the last glacial maximum 20 Kya [26].

While PSMC allows inference of pig demography up to 1 Mya, it does not provide information on recent (10 Kya to current) demography. However, insight into more recent demography can be obtained from analysis of runs of homozygosity (ROH) in the genome. In particular large ROH are most sensitive to recent population changes. A comparison between European and Asian wild boars showed an on average much larger number of ROH in European wild boars, with clear indications of recent inbreeding [12], which is in agreement with the strong recent bottlenecks that were caused by over-hunting and a decline in suitable habitats in Europe.

The severe population bottleneck during the last glacial period and recent inbreeding resulted in much lower genetic diversity in European wild boar compared to Asian wild boar [8]. This lower genetic diversity might also be explained by a bottleneck due to migration of wild boar from Asia to Europe, although no indication for such a bottleneck is evident from the PSMC analysis [8]. While this difference in genetic diversity is also observed for European and Asian domestic breeds, the recent gene flow from Asian domestic into European domestic breeds [41] resulted in higher nucleotide diversity in European breeds than in the European wild boar (discussed below) [8, 12, 20]. In agreement with these observations, approximate Bayesian computation by [20] showed an expected (modest) decline in effective population size in Asian domestic pig breeds compared to Asian wild boar, while the effective population size in European domestic pig breeds was similar to that of Asian domestic breeds and more than twice as large as that of European wild boar.

### Pig domestication

Of all the suids, only wild boars have managed to spread across several continents, which shows that they are extremely adaptable to a wide range of environments and climates. In that regard, it is interesting that wild boar appears to have been the only suid that was domesticated by humans. The latter might be simply due to its wide distribution across Eurasia, bringing them into frequent direct contact with humans, but it is also possible that the extreme adaptability of this species has contributed to its domestication by humans. Domestication of the pig took place some 9000 to 10,000 years ago independently at two locations: in East Anatolia and in China. Following these initial domestications, pigs accompanied early farmers as they spread from East Anatolia to Europe and throughout China, respectively. The picture that has emerged from analysis of pig mitochondrial genomes [42, 43] and more recently from whole-genome sequence data [20] is that this domestication process was very diffuse, taking many millennia and involving repeated admixture and gene flow from wild boars into the domesticated populations. Therefore, pig domestication should not be considered as a series of fixed events that happened some 10,000 years ago but as a gradual process, in which both animal and humans played their part. Wild boars might have been initially attracted to human settlements as an easy way of accessing food, and it is only after millennia that humans might have actually started to keep pigs as a truly domesticated species. This process occurred in Europe and China at vastly different rates, with pigs being kept in enclosures within human settlements at a relative early stage in China, while in Europe until the late middle ages, pigs were allowed to freely roam the forests as domesticated herds [44]. By the late middle ages, European and Asian domestic pigs were genetically very different for two reasons: (1) they were based on wild boar populations that diverged around 1 Mya ago and differed at over one million positions throughout their genomes [8]; and (2) for thousands of years they were submitted to selection pressures on very different traits. Nevertheless, it is expected that, at the same time, selection acted on similar traits such as behaviour (docility) and morphology (such as coat colour and body size). Indeed, early studies on pooled genome sequence data suggest that selection occurred on genes that affect such traits [37, 45]. Genomic regions and genes that are likely to have been under selection will be discussed in the next section, but there is one other event that played a major role in shaping the genomes of the majority of modern domestic pig breeds. By the late eighteenth- to early nineteenth-century, pig breeding, in particular in the UK, underwent a series of major changes due to growing demands for pig meat as a result of growth of the human population at the time of the industrial revolution. Breeders turned their attention to Asia and imported Chinese pigs to improve their breeding stock [41]. Admixture analysis using D-statistics [46] indicates 35 % Asian contributions to the genomes of modern European commercial breeds [8, 14, 20], while haplotype-based estimates [16, 47] and a partially supervised admixture analysis [48] suggest a 20 % Asian contribution in European domestic pigs. In agreement with historical records, Iberian pig breeds show no signs of admixture with Chinese pigs [13, 48].

### Identification of selective sweeps in the pig genome

Domestication and subsequent selection by humans have generated an enormous amount of phenotypic variations that are not seen in the original wild animals. Current pig breeds in Europe and Asia exhibit a vast range of distinct morphological characteristics related to, e.g. body size, coat colour, ear shape, and shape of the skull. Other phenotypes related to reproduction and behaviour of these animals have also changed dramatically compared to their wild progenitors. To understand the molecular mechanisms and to identify the genes that underlie these changes, it is important to distinguish changes during early domestication from more recent changes during breed development and intense selection by breeders. Many of the observed morphological and behavioural changes seen in pigs are also observed in other domestic species [49, 50], which suggests that selection occurred on genes within the same pathways that affect these biological processes in these species. Several of these morphological changes, such as coat colour (spots), floppy ears, and curly tails, have been suggested to be directly correlated with domestication and selection for tameness [50]. Nevertheless, it is likely that many of these characteristics (in particular coat colour) were favoured by some breeders and were strongly selected for in certain breeds after initial domestication. Several mutations with a major effect on coat colour (*KIT* [51], *MC1R* [52]) or lean growth (*IGF2* [53], *RYR1* [54], *PRKAG3* [55]) have been identified. While providing examples of genes that clearly have been under strong selection, the number of genes identified remains small, and they do not provide insight into the changes within the genome during initial domestication of pigs.

The possibility to sequence the genome of multiple individuals has enabled the decision to start to address this in more detail. This has resulted in several studies that used whole-genome sequence data to identify selective sweeps in pigs and numerous methods have been developed to identify such signatures of selection [56]. It is important to realize that different models underlie these methods and, thus, interpretation of the results must be done with caution. Genomic evidence for selection based on these analyses is often suggestive rather than conclusive, and it is often difficult to distinguish drift from true selection. One of the first studies that used next-generation sequence data from populations towards this aim was based on reduced representation sequencing data that represented only 2 % of the genome [45]. The results from that study suggested selection on genes that affect coat colour, growth, muscle development, olfaction, immunity, and brain development. Among the identified regions, were those that harbour potential candidate genes affecting behaviour, such as the *PPP1R1B* gene on pig chromosome (SSC for *S. scrofa*) 12 and the *LRRTM* gene on SSC2. However, this study lacked sufficient resolution to unequivocally identify specific genes due to the low coverage of the sequence data. The first study that used sequence data covering the complete genome of multiple individuals compared genome sequence data that were generated on pools of European domestic and European wild boars [37]. This approach identified several strong candidate genes that have been under selection and that affect body size (*NR6A1*, *PLAG1*, and *LCORL*) or body composition (*OSTN*). Furthermore, these authors also identified 72 derived nonsynonymous substitutions that approached fixation in domestic pigs, including the Pro192Leu missense mutation in *NR6A1*, which is most likely the causative mutation that resulted in the increased number of vertebrae in domestic pigs. These authors also revealed the staggering complexity of multiple duplications around the *KIT* gene and its potential regulatory sequences, which are responsible for different coat colour phenotypes, such as dominant white, patch, and belt. This provides further evidence that structural changes, many of which affect *cis*-acting regulatory sequences, underlie the observed rapid evolution in domestic animals [57].

Using a combination of genotyping data for 60 K SNPs on a large number of individuals and genome sequence data for a limited number of the individuals, Wilkinson et al. [58] were able to identify a region on SSC1 with evidence of differential selection in domestic breeds compared to wild boar. This selection signal is within a region that contains the *THSB2* and *SMOC2* genes, which have been suggested to affect skull development in dogs [59, 60], thus, they are potential candidates for genes that have been under selection at an early stage during domestication. A number of studies identified selective sweeps in specific breeds, such as the Berkshire breed [21, 25] and the Yucatan minipig [19], and suggested that selection on genes that affect growth and fatness traits occurred in these breeds. However, it is likely that all these represent genes that have been under selection very recently during the formation of these breeds. Furthermore, it remains unclear how many of these genes have only been under selection in the specific populations studied or whether these signals are the result of drift in these populations.

The different origin of the Chinese and European breeds has resulted in many fixed sequence differences in their genomes and distinct sequence variants have been selected for in the European versus the Asian breeds [8]. Examples of selective sweeps of European variants are the sweeps around the *NR6A1*, *PLAG1* and *LCORL* genes [37] described above. A number of studies specifically analysed selective sweeps in Asian pigs [16, 17, 23]. In a study [18] that included the Korean native pig breed, selective sweeps were observed around the *CLDN1* and *TWIST1* genes, which may affect fertility and fatness, respectively. Asian breeds are well known for their high prolificacy, fertility and fatness. Strong selective sweep signals at genes with a potential effect on fertility (*GPR149* and *JMJD1C*) and on genes that are involved in fatness traits were identified in the Chinese Tongcheng breed [23]. The same authors reported that the *MTF* and *EDNRB* genes were responsible for the coat colour phenotype of this breed, which is sometimes referred to as the Huazhong Two-End Black pig. These findings confirm earlier ones on the involvement of the *EDNRB* gene in the coat colour phenotype of Chinese spotted pigs [16] and of the old English breed Gloucester Old Spot [58]. The *EDNRB* variant that was identified in the Gloucester Old Spot breed was shown to be of Chinese origin and to result from the introgression of Chinese pigs into European breeds during the early nineteenth century. Another Chinese variant that was selected for in European breeds after its introgression is the region around the *LEMD3* gene [58], which shows a strong selection signature that is associated with ear morphology (floppy vs. upright ears). Bosse et al. [14, 61] subsequently performed a selective sweep analysis that was specifically designed to identify Asian haplotypes that have been under strong selection after their introgression into European breeds in the early nineteenth century. In agreement with the distinct features that differentiate Asian and European pigs with regard to a number of morphological traits, fertility and fatness, selective sweeps in regions that harbour genes affecting these traits were identified. This specific approach allowed the authors to identify Asian-derived non-synonymous mutations in the *AHR* gene on SSC9 that is associated with increased litter size [14]. These studies also highlighted regions that specifically lacked introgressed Asian haplotypes in the Large White population studied [61], several of which contained genes that are known to have been under selection for European variants (*MC1R*, *KIT*).

With the possible exception of the selective sweep around the *THSB2* and *SMOC2* genes, the genes described above were most likely under selection long after the initial stages of domestication. The traits that are most likely to have been under selection during early domestication are behaviour and increased tameness. Therefore, genes that are involved in brain and neuronal development are likely candidates to have been under selection during this process. Indeed, several studies [20, 23, 25, 45, 62] reported overrepresentation of genes with GO (gene ontology) terms related to neuronal development and neurological regulation, although care must be taken to avoid over-interpreting the results of such GO analyses. Moreover, the selective sweeps identified in these studies concerned different locations and genes and there was no gene that was consistently identified in multiple studies. Nevertheless, this could also be related to the complex genetic background of traits such as behaviour and increased tameness, as suggested in a study that addressed domestication genes in the rabbit [63]. These authors suggested that in rabbit and other domesticated animals, selection for these traits involved allele frequency shifts at many loci, rather than at a few major domestication loci. Frantz et al. [20] investigated whether parallel selective sweeps at the same loci had occurred in Asian and European domestic pigs. Whereas they did find several such loci, the majority were attributed to the introgression of Asian alleles into European breeds in the nineteenth century. Nevertheless, they identified one locus around position 82.37 Mb on SSC4 (build10.2) that had been independently selected for in European and Asian pigs, although it is located 70 kb from the nearest annotated gene (*PENK*). The lack of annotated genes in the pig genome at this location does not appear to be attributable to the draft state of the pig reference genome because the homologous region in the human genome is also a gene desert, at least with respect to protein-coding genes.

## Concluding remarks

A large variety of genes have been identified and implicated as being under strong selection in pigs. Some of these genes exhibit a strong effect and were identified in many studies. Variants of these genes have also been implicated as being under strong selection in other species, which further supports their involvement during selection in pigs. Still, distinguishing true selection from drift remains a challenge that requires further studies. However, so far these studies have clearly shown the importance of a well-designed comparison that includes well-characterized populations and takes historical information on the populations into account. A good example is the well-known and well-documented introgression of Asian haplotypes into European pigs in the late eighteenth- to early nineteenth-century.

However, analysis of DNA samples from current animals will always be limited in what it can teach us about past events. Recent progress in obtaining whole-genome sequence data from ancient DNA samples [22] extracted from fossils, will undoubtedly further revolutionize our ability to more directly provide insight into historical events [43, 64] and to reconstruct selective sweeps in these populations at high resolution.

Although the availability of a pig reference genome has enabled analysis at a resolution not possible before, the draft status of the reference genome hinders or even prevents analysis at many loci in the pig genome [35]. Furthermore, a large number of selective sweeps has been identified at regions that lack annotated genes and many of these regions most likely harbour important regulatory sequences that affect nearby genes. The draft status and incomplete annotation of build 10.2 of the pig reference genome are also highlighted by the recently published improved assembly of the porcine X and Y chromosomes [65]. A comparison between build 10.2 and the new chromosome X assembly reveals many rearrangements in the former and the total number of annotated genes increased from 632 in build 10.2 to 1033 in the new build. Clearly, to fully benefit from the wealth of whole-genome sequence data that continues to be generated, an improved reference genome and improved annotation [66] for all pig chromosomes are essential prerequisites to fully capitalize on this wealth of information.
